# Characterization of the enzyme kinetics of EMP and HMP pathway in *Corynebacterium glutamicum*: reference for modeling metabolic networks

**DOI:** 10.3389/fbioe.2023.1296880

**Published:** 2023-11-28

**Authors:** Liu Yang, Junyi Li, Yaping Zhang, Linlin Chen, Zhilin Ouyang, Daocheng Liao, Fengguang Zhao, Shuangyan Han

**Affiliations:** ^1^ Guangdong Key Laboratory of Fermentation and Enzyme Engineering, School of Biology and Biological Engineering, South China University of Technology, Guangzhou, China; ^2^ School of Light Industry and Engineering, South China University of Technology, Guangzhou, China

**Keywords:** *Corynebacterium glutamicum*, EMP pathway, HMP pathway, enzyme kinetics parameters, metabolic network models

## Abstract

The model of intracellular metabolic network based on enzyme kinetics parameters plays an important role in understanding the intracellular metabolic process of *Corynebacterium glutamicum*, and constructing such a model requires a large number of enzymological parameters. In this work, the genes encoding the relevant enzymes of the EMP and HMP metabolic pathways from *Corynebacterium glutamicum* ATCC 13032 were cloned, and engineered strains for protein expression with *E.coli* BL21 and *P.pastoris* X33 as hosts were constructed. The twelve enzymes (GLK, GPI, TPI, GAPDH, PGK, PMGA, ENO, ZWF, RPI, RPE, TKT, and TAL) were successfully expressed and purified by Ni^2+^ chelate affinity chromatography in their active forms. In addition, the kinetic parameters (*V*_max_, *K*_m_, and *K*_cat_) of these enzymes were measured and calculated at the same pH and temperature. The kinetic parameters of enzymes associated with EMP and the HMP pathway were determined systematically and completely for the first time in *C.glutamicum*. These kinetic parameters enable the prediction of key enzymes and rate-limiting steps within the metabolic pathway, and support the construction of a metabolic network model for important metabolic pathways in *C.glutamicum*. Such analyses and models aid in understanding the metabolic behavior of the organism and can guide the efficient production of high-value chemicals using *C.glutamicum* as a host.

## 1 Introduction

Cellular metabolism involves the conversion of one substance into another through a series of enzymatic reactions (Feierabend et al., 2021). By collecting and summarizing data on these enzymes and simulating metabolic networks involved, we can achieve a better understanding of dynamic behavior of cellular system, elucidate metabolic pathways flux distribution, and guide metabolic engineering design in the yield of potential targets or metabolites (Albert, 2007; Covert et al., 2001; Gombert and Nielsen, 2000). Altintas MM (Altintas et al., 2006) has used the published values for kinetic parameters of individual enzymatic reactions in the pentose phosphate pathway (HMP pathway) and the Entner-Doudoroff (ED) pathway to develop an intracellular metabolic network model in *Aspergillus oryzae*. They conducted successfully *in silico* simulations to enhance xylose utilization and increase the yield of desired metabolites.

However, it is worth noting that even for networks databases as extensive as KEGG or MetaCyc, the available parameters often prove insufficient to construct precise metabolic model and describe metabolite dynamics. An important reason for this limitation is that these parameters are typically obtained or estimated from measurements reported by different laboratories, each employing various *in vitro* models and conditions (Tohsato et al., 2013). Bertilsson et al. (2008) compiled kinetic parameters of enzymes from various sources, constructed a kinetic model for glucose and xylose co-substrates uptake in *Saccharomyces cerevisiae*, and proposed strategies to improve co-substrate utilization based on transport systems modifications. However, the kinetic parameters for Hxt3p with respect to xylose were unavailable in the literature, requiring researchers to relay on parameters from another homologous enzyme, potentially affecting the accuracy of the model. Dräger used multiple kinetic equations and presented a comprehensive mathematical model of valine and leucine biosynthesis in *C*. *glutamicum* (Drager et al., 2009). Nevertheless, kinetic modeling or metabolic network reconstitution necessitates an extensive amount of information regarding the kinetics of each enzyme, including reaction rates, Michaelis constants, and so on (Andersen and Nielsen, 2009; Zupanic et al., 2020).

*Corynebacterium glutamicum* has been engineered as an important platform organism which far outweighed amino acid production during the last decades. Notably, it has not only excelled in producing organic acids such as pyruvic acid and succinic acid, but also demonstrated the capability to synthesize high-value compounds like putrescine (1,4-diaminobutane) and resveratrol (Kallscheuer et al., 2019; Kogure et al., 2016; Zha et al., 2023). In silico, well-characterized metabolic modeling is expected to enable the design of artificial metabolic networks for desired product synthesis in *C.glutamicum*. Hence, it is essential to have accurate data regarding some central metabolism or related pathways, including glycolysis (EMP pathway) and the pentose phosphate pathway (HMP pathway) since they provide ATP as well as offer reducing power (NADPH) in cells (Aziz and Mohiuddin, 2022; Tan et al., 2016). The acquisition of comprehensive and systematic kinetic parameters for enzymes involved in these pathways will undoubtedly facilitate the construction of intracellular metabolic networks and enhance the accuracy of mathematical models.

In this study, the EMP and HMP pathway-related enzyme genes were cloned from the model strain *C*.*glutamicum* ATCC13032. Subsequently, these genes were expressed exogenously in *Escherichia coli* BL21 (DE3) or *Pichia pastoris* X33, and the corresponding enzymes were purified and prepared as far as possible. By determining and calculating the kinetic parameters of these enzymes, valuable data for the construction of a metabolic network model encompassing the central metabolic pathways in *C. glutamicum* were obtained. In order to provide a more comprehensive reference for researchers, we also collected and summarized the enzymatic properties of reported enzymes related to the above two pathways from different cells. These works contribute significantly to a thorough understanding of the dynamic behavior of cellular systems and flux control distribution in *C. glutamicum*. As a result, they greatly benefit the design of new metabolic pathway in *C. glutamicum*, positioning it as a promising cell factory for potential application.

## 2 Material and methods

### 2.1 Strains and culture conditions

The plasmid of pET-28a (+): Bacterial vector for the expression of N-terminal 6x His-tagged proteins. *E.coli* TOP10F′ cells (Invitrogen, Carlsbad, CA, United States) were cultured at 37°C in Luria-Bertani medium (LB) (1% w/v tryptone, 0.5% w/v yeast extract, 1% w/v sodium chloride) containing 50 mg/L kanamycin to select and obtain the recombinant plasmid. *E.coli* BL21 (DE3) served as host for heterologous gene expression and was grown in LB medium at 37°C and 200 rpm. *P.pastoris* X33 was used as host for heterologous gene expression and grown in BMMY/BMGY medium at 30°C and 250 rpm. These cells were used for DNA manipulations. Buffered methanol complex and glycerol complex medium (BMMY/BMGY) was prepared according to yeast fermentation guidelines.

### 2.2 Cloning of EMP and HMP pathway related enzyme genes from *C*. *glutamicum* ATCC 13032

**FIGURE 1:**
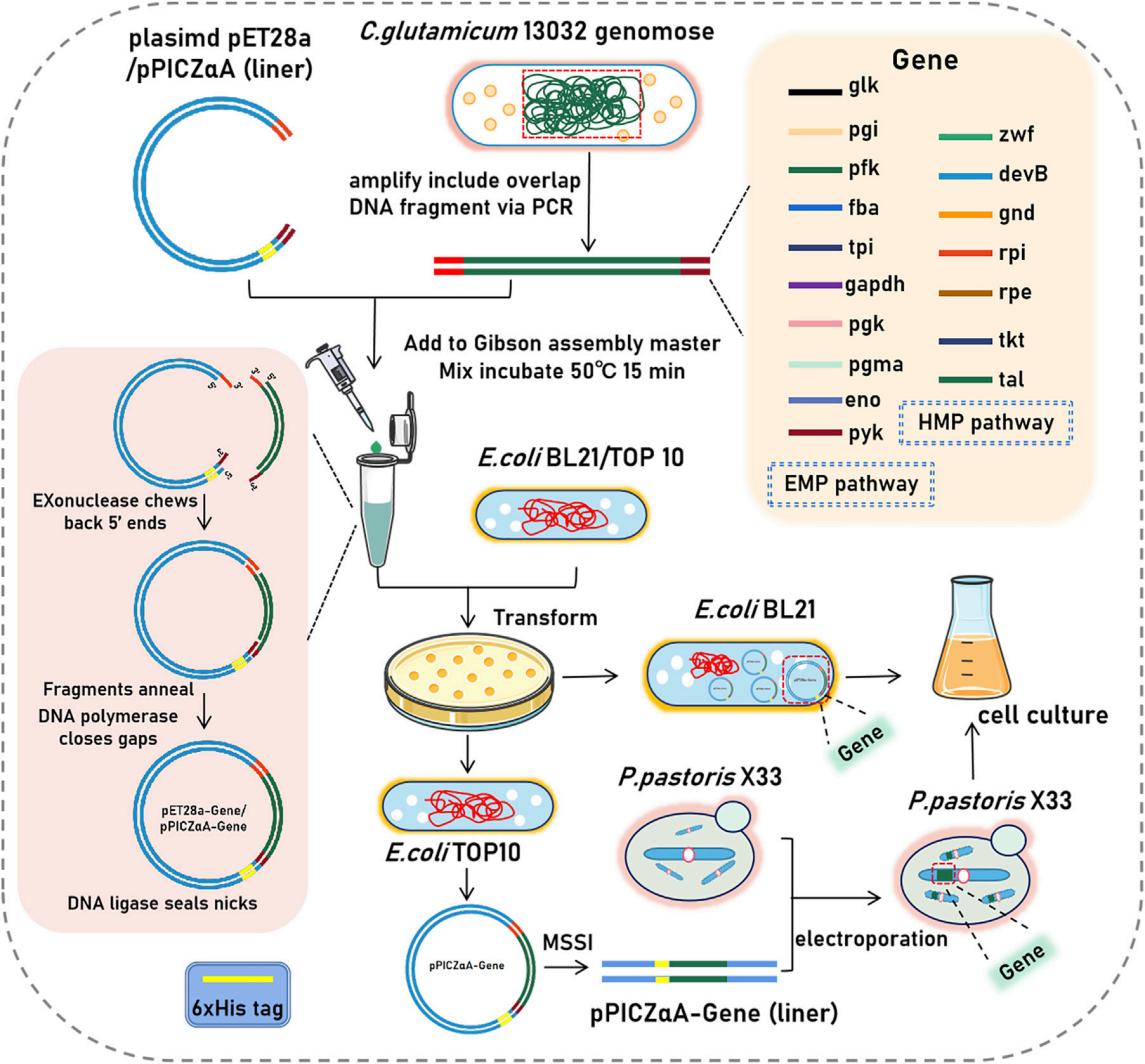
Schematic diagram of plasmid and expression strains construction. (*glk*, glucokinase; *gpi*, glucose-6-phosphate isomerase; *pfk*, 6-phosphofructokinase; *fba*, fructose-bisphosphate aldolase; *tpi*, triosephosphate isomerase; *gapdh*, glyceraldehyde 3-phosphate dehydrogenase; *pgk*, phosphoglycerate kinase; *pgma*, phosphoglycerate mutase; *eno*, enolase; *pyk,* pyruvate kinase; *zwf*, glucose-6-phosphate dehydrogenas; *gnd,* gluconate 6-phosphate dehydrogenase; *devB*, 6-phosphogluconolactonase; *rpi*, ribose-5-phosphate isomeras; *rpe*, ribulose-5-phosphate 3-epimerase; *tkt*, transketolase; *tal*, transaldolase).

Seventeen enzyme genes which related to EMP and HMP pathway from the genomic DNA of *C.glutamicum* ATCC 13032 (GeneBank: GCA_000011325.1) were clonded. The primers used for amplification were designed based on the nucleotide sequences of these genes (Supplementary Tables S1, S2). The coding sequence of the related protein genes were inserted into the expression plasmid of pET28a (+) by using homologous recombination technology, and their sequences were verified by DNA sequencing. The plasmids are then transformed into *E.coli* BL21 (DE3) for expression (Figure 1).

In addition, five enzyme genes (6-phosphofructokinase, fructose-bisphosphate aldolase, enolase, pyruvate kinase, and gluconate-6-phosphate dehydrogenase) were inserted into the expression plasmids of pPICZαA. Similar to pET28a (+) plasmids, the constructed pPICZαA plasmids was also verified by DNA sequencing. To enable expression in *P.pastoris* X33, the pPICZαA plasmids were linearized using a restriction enzyme digestion site *MSSI* on the plasmid. The linearized plasmids were then introduced into *P.pastoris* X33 cells via electroporation (Feng et al., 2017).

### 2.3 Enzyme expression in *E.coli* BL21 and *P.pastoris* X33

To prepare the inoculum, 100 μL of the cell suspension stored at −80°C was transferred to a 50 mL Erlenmeyer flask containing 10 mL of LB medium with 50 mg/L kanamycin, incubated at 37°C and 200 rpm for 10–12 h, and inoculated at a ratio of 1:100 (v/v) in 100 mL shake flasks at 37°C and 200 rpm until the OD_600_ nm values in the LB medium reached 0.6–0.8 (Carmignotto and Azzoni, 2019). Isopropyl-β-D-thiogalactoside (final IPTG concentration 0.4 mM) was then added to the culture medium and incubation continued for another approximately 16 h at 16°C. Cells were collected at the end of induction by centrifugation at 6,000 rpm, 15 min, 4°C.

Inoculums were prepared by transferring 100 μL of the cell suspension stored at −80°C to a 50 mL erlenmeyer flask containing 5 mL of BMGY medium with 100 μg/mL zeocin and supplemented with 1% (v/v) glycerol as the sole carbon source. Culture was performed overnight at 30°C and 250 rpm. The inoculum was then inoculated into 250 mL shake flasks containing 50 mL of BMMY medium until the OD_600_ nm values in the BMMY medium reached 1.0, and supplemented with 1% (v/v) methanol as the sole carbon source, incubation was continued for another 3–5 days at 30°C. The supernatant was obtained at the end of cultivation by centrifugation at 6,000 rpm, 10 min, 4°C.

### 2.4 Enzyme purification and analysis of protein concentration

The collected cells were resuspended in 10 mL of buffer A (20 mM Tris-HCl, pH 7.0, and 500 mM NaCl). This was followed by comminution by ultrasonication in an ice-water bath with 300 cycles of 3 s of sonication followed by a 3-s pause between cycles. The crude extract (supernatant) was obtained by centrifugation (10,000 rpm, 20 min, 4°C), and enzymes associated with the EMP and the HMP pathway were purified by a nickel ion affinity chromatography column and analyzed by SDS-PAGE. For analysis SDS-PAGE samples were taken before induction, after induction, the crushed supernatant and the precipitate to analyse the solubility and molecular weights of the protein. Gels were stained with Komas Brilliant Blue R-250 and decolorized by washing with 10% acetic acid. Enzyme concentrations were determined by the Bradford method using bovine serum albumin as a concentration standard (Xue-ling et al., 2010).

The supernatant was filtered through a 0.22 µm membrane and purified with a nickel-affinity chromatography column (TIANGEN Biotech (Beijing) Co.). The column was equilibrated with buffer A and the purification pathway was rinsed. The heteroproteins were first eluted with buffer B (0.5 M imidazole, 20 mM Tris-HCl pH 7.0, and 500 mM NaCl) containing 0.5 M imidazole, and then each enzyme was eluted in turn with buffer B containing 0.2 M imidazole, 0.3 M imidazole and 0.5 M imidazole. The purified samples were desalted in a desalting column with buffer A.

### 2.5 Determination of enzymatic kinetic parameters

Enzyme kinetic parameters were measured in the 100 mM PBS buffer (137 mM NaCl, 2.7 mM KCl, 10 mM Na_2_HPO_4_, 2 mM KH_2_PO_4_, 0.5 mM MgCl_2_, pH7.4) and at 37°C (Supplementary Material S2). One unit (U) of enzyme activity was defined as the amount of enzyme that catalyzes the conversion of 1 µmol of substrate per minute into specific products (Tan et al., 2016). With substrate concentration as the horizontal coordinate and enzyme reaction rate as the vertical coordinate, the curve of the Mee equation was fitted using origin software. From the fitted cureve, the values of *K*_m_ (Michaelis constant) and *V*_max_ (maximum reaction rate) were calculated. Furthermore, the turnover number, *K*_cat_, was calculated based on *K*_cat_ = *V*_max_/enzyme concentration.

## 3 Results

### 3.1 Expression of EMP and HMP pathway related enzymes

**FIGURE 2:**
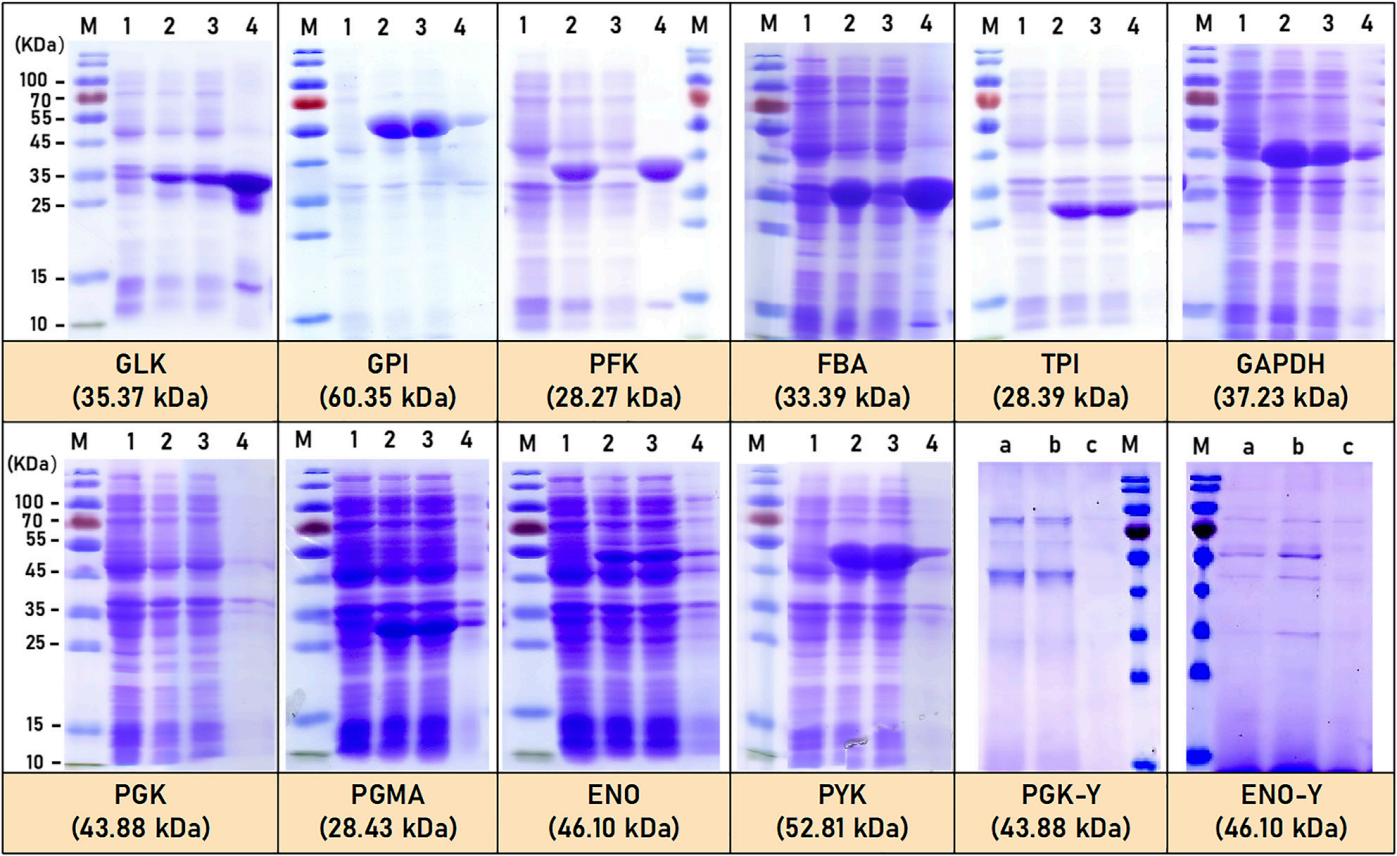
SDS-PAGE of EMP pathway related enzymes expression by *E.coli* and *P.pastoris*. M: marker; 1: before induction; 2: after induction; 3: crushed supernatant; 4: precipitate; a: yeast expression for 3 days; b: yeast expression for 5 days; c: control.

Six enzymes (GLK, GPI, TPI, GAPDH, PGMA, and PYK) from the EMP metabolic pathway of *C*.*glutamicum* ATCC 13032 were successfully expressed exogenously in *E.coli* BL21. SDS-PAGE analysis confirmed that all six enzymes were soluble, and their molecular weights are found to be consistent with the reported or predicted values in the literature (Mathur et al., 2005; Mathur et al., 2006; Sangolgi et al., 2016a). The theoretical molecular weights of these enzymes are estimated to be 35.37 kDa (GLK), 60.35 kDa (GPI), 28.39 kDa (TPI), 37.23 kDa (GAPDH), 28.43 kDa (PGMA), and 52.81 kDa (PYK), separately (Figure 2). However, two enzymes (PFK and FBA) were expressed as inactive inclusion bodies form. The other two enzymes (PGK and ENO) that could not expressed in *E.coli* BL21 were successfully expressed exogenously in *P*.*pastoris* X33. The molecular weight of the expressed PGK and ENO enzymes in *P. pastoris* X33 are closed to the reported or predicted values in the literature (Cayir et al., 2014a; Shang et al., 2021), which are predicted to be 43.88 kDa (PGK-Y) and 46.10 kDa (ENO-Y), respectively (Figure 2).

**FIGURE 3:**
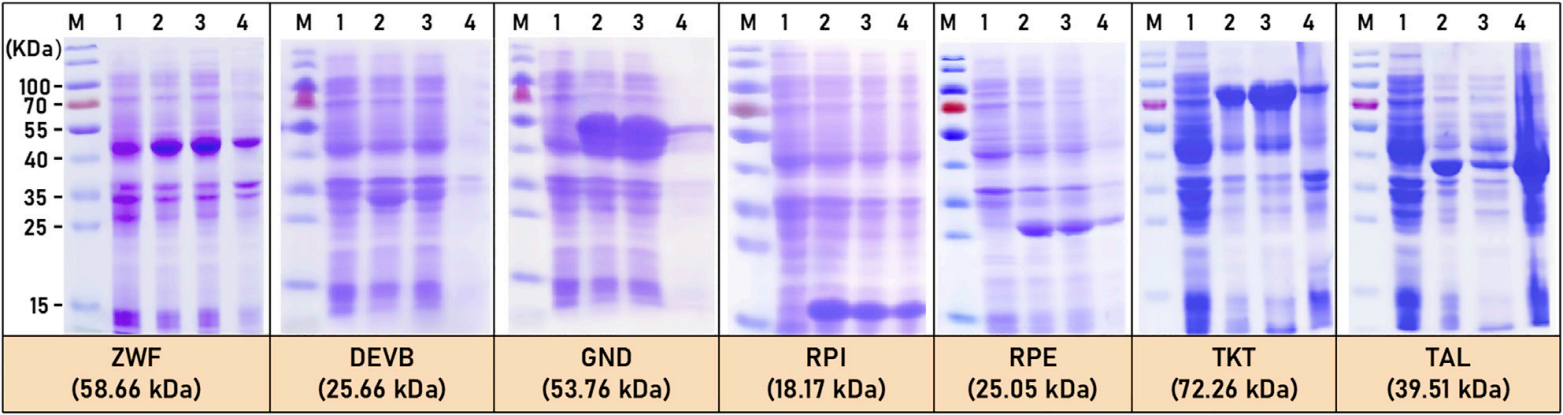
SDS-PAGE of HMP pathway related enzymes expression. M: marker; 1: before induction; 2: after induction; 3: precipitate; 4: crushed supernatant.

On the other hand, all seven enzymes (ZWF, DEVB, GND, RPI, RPE, TKT, and TAL) of the HMP pathway in *C*.*glutamicum* ATCC13032 were successfully expressed exogenously in *E.coli BL21*. SDS-PAGE analysis confirmed that all seven enzymes are soluble proteins, with only a small amount observed as inclusion bodies (Figure 3). Importantly, the molecular weight positions of the expressed enzymes closely matched the predicted values. Specifically, the molecular weights of seven enzymes were predicted as follows: ZWF (58.66 kDa), DEVB (25.66 kDa), GND (53.76 kDa), RPI (18.17 kDa), RPE (25.05 kDa), TKT (72.26 kDa), and TAL (39.51 kDa) (Figure 3).

### 3.2 Purification of enzymes associated with the EMP and HMP pathway

**FIGURE 4:**
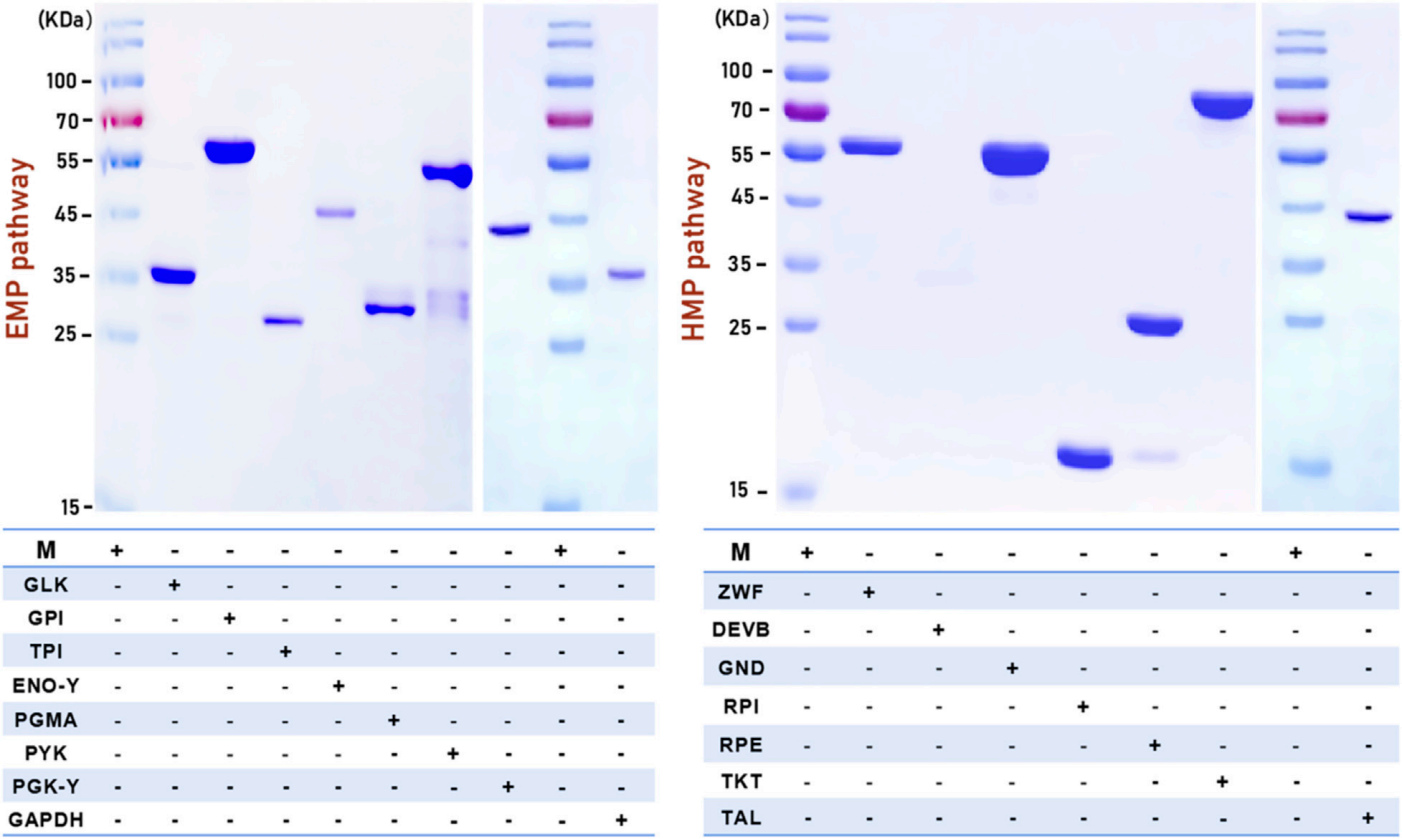
SDS-PAGE of EMP and HMP pathway related pure enzymes. “**+**“: contain related enzyme in this swimming lane; “-“: excluding related enzyme in this swimming lane.

The fifteen successfully expressed enzymes from crude cell extracts were purified using Ni ion-affinity chromatography columns, which utilized a His tag at either the C-terminus or N-terminus of the recombinant enzymes. Validation of the purified samples by SDS-PAGE showed that GLK, GPI, TPI, GAPDH, PGMA, ENO, PYK, PGK-Y, and ENO-Y were eluted with buffer B containing 0.5 M, 0.2 M, 0.2 M, 0.3 M, 0.3 M, 0.3 M, 0.3 M, 0.2 M, and 0.2 M (elution concentration of protein) imidazole, respectively. ZWF, GND, RPI, RPE, TKT and TAL were eluted with buffer B containing 0.5 M, 0.5 M, 0.5 M, 0.3 M, 0.3 M and 0.2 M imidazole, respectively (Figure 4).

### 3.3 Determination of kinetic parameters of enzymes associated with the EMP and HMP pathways

**FIGURE 5:**
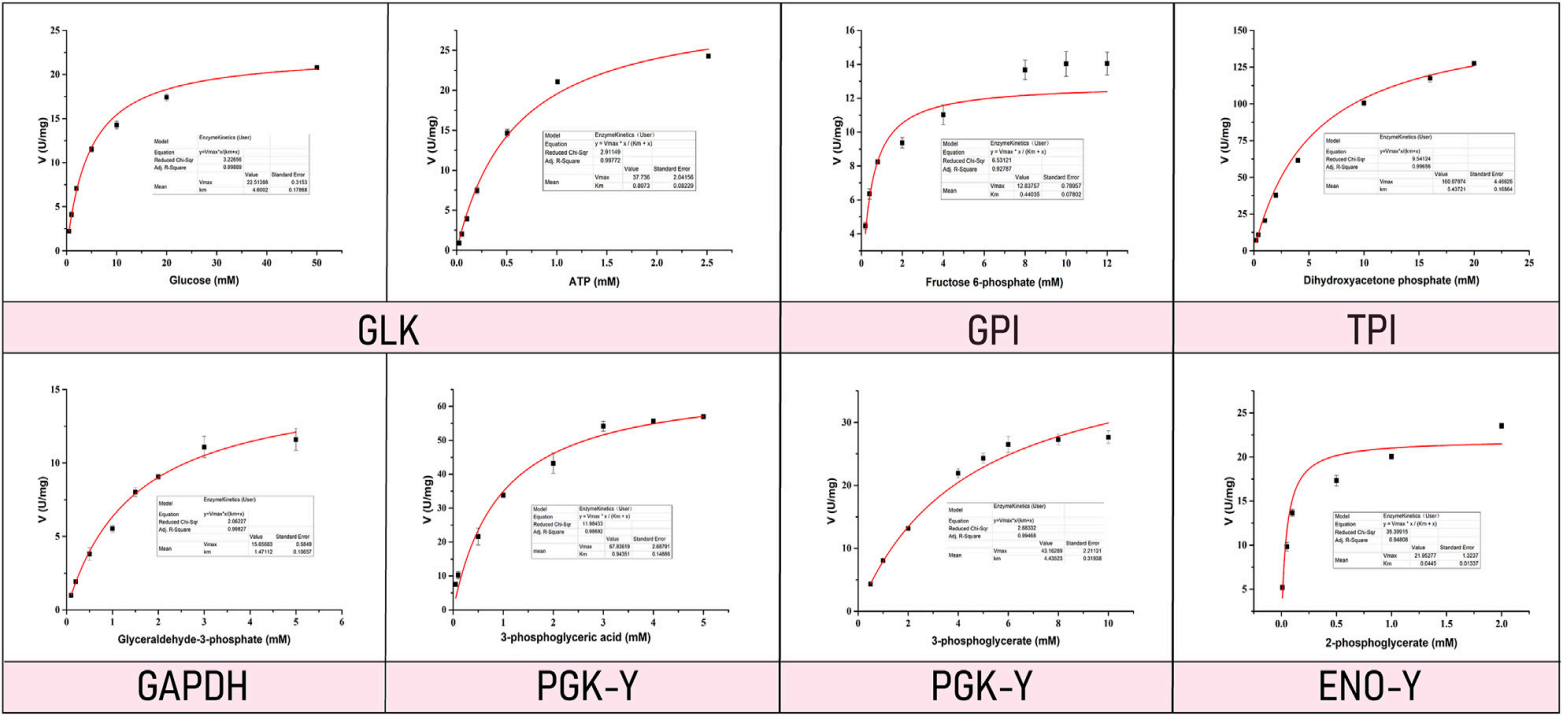
Enzyme kinetic curve of EMP pathway related enzymes.

**FIGURE 6:**
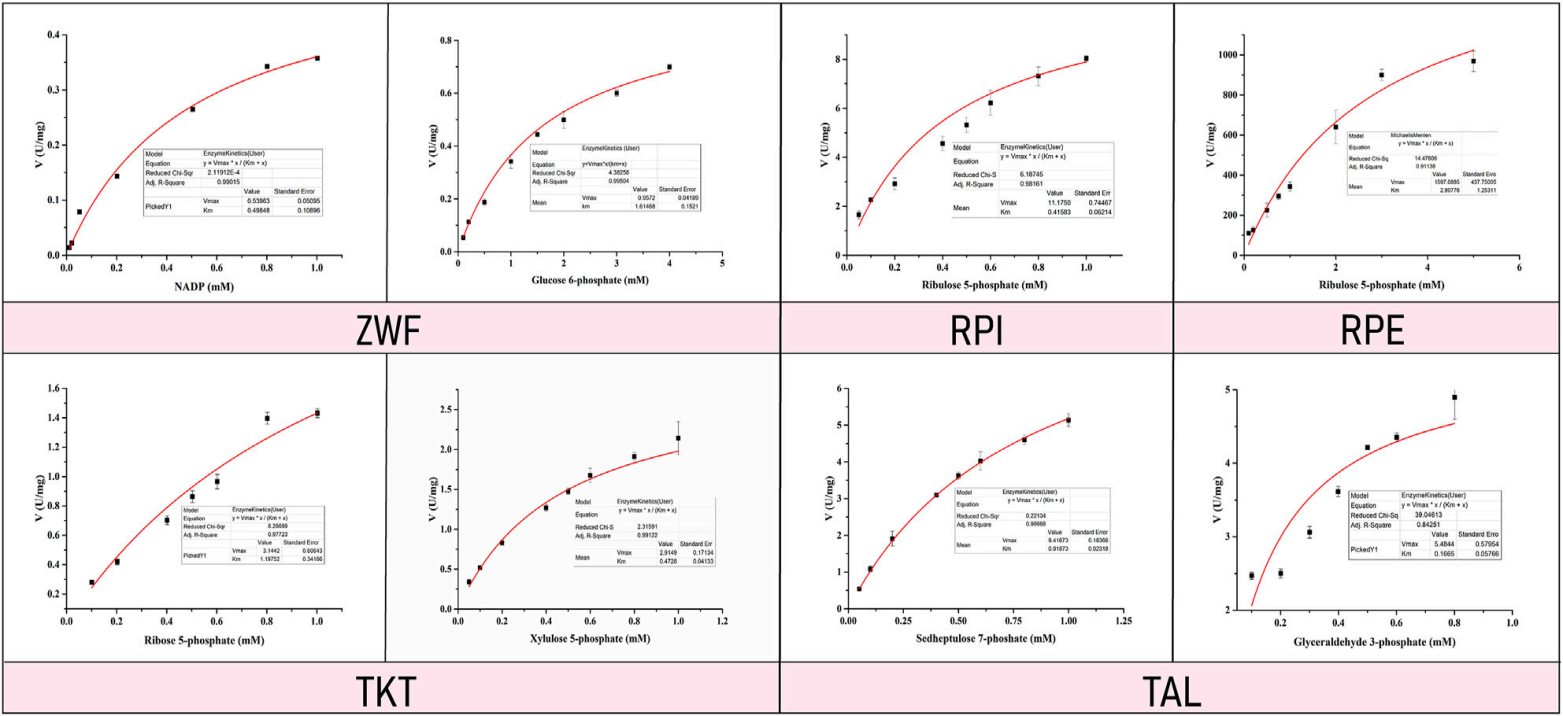
Enzyme kinetic curve of HMP pathway related enzymes.

Enzyme kinetic curves were obtained by conducting experiments (Figures 5, 6), and enzyme kinetic parameters were calculated for fourteen purified enzymes from the EMP and HMP pathways (Table 1). The kinetic measurements were carried out under consistent conditions, ensuring the temperature and pH remained constant. However, it was observed that the purified enzymes PYK and GND did not show any activity with their respective substrates, namely phosphoenolpyruvate and glucose-6-phosphate. Additionally, the substrate for DEVB could not be determined as it was not available or provided in the previous studies and literature.

**TABLE 1:** Collection of enzyme kinetic parameters of EMP, HMP pathway-related enzymes.

Enzyme	Substrate	Organism	Expression host	*K*_m_ (mM)	*V*_max_ (U mg^-1^)	*K*_cat_ (s^-1^)	References
GLK	Glucose 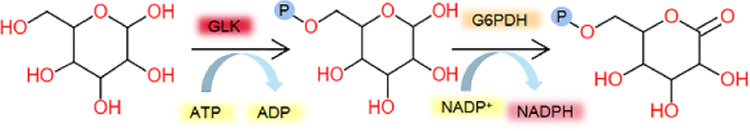	*C.glutamicum*	*E.coli*	4.60 ± 0.18	22.51 ± 0.31	13.13 ± 0.16	This study
		*N*. *fowleri*	*E.coli*	0.042 ± 0.0073	-	36.50	Milanes et al. (2019)
		Human liver	*E.coli*	-	-	33.20 ± 0.60	Jiang et al. (2021)
	ATP 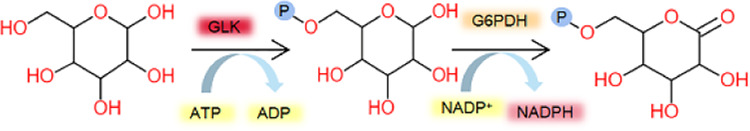	*C.glutamicum*	*E.coli*	0.81 ± 0.08	37.74 ± 2.04	22.24 ± 1.07	This study
		*N*. *fowleri*	*E.coli*	0.14 ± 0.0099	-	36.50	Milanes et al. (2019)
		Human liver	*E.coli*	1.00 ± 0.24	-	43.80 ± 3.90	Jiang et al. (2021)
GPI	D- fructose-6-phosphate 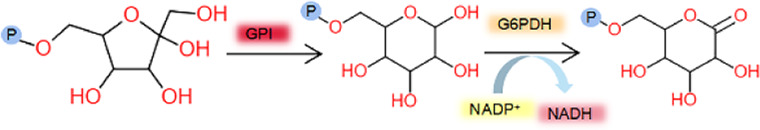	*C.glutamicum*	*E.coli*	0.57 ± 0.13	14.04 ± 0.66	141.20 ± 4.52	This study
		*M*.*jannaschii*	*E.coli*	0.04	21.00	-	Rudolph et al. (2004)
TPI	Dihydroxyacetone phosphate 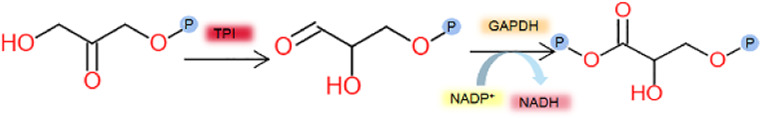	*C.glutamicum*	*E.coli*	5.43 ± 0.16	160.08 ± 4.47	75.74 ± 2.11	This study
		Fasciola hepatica	*E.coli*	2.30 ± 0.25	-	25,000.00 ± 1200.00	Zinsser et al. (2013)
GAPDH	Glyceraldehyde triphosphate 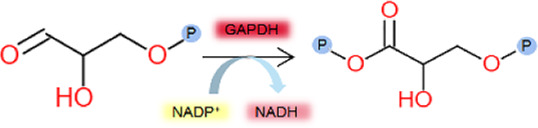	*C.glutamicum*	*E.coli*	1.47 ± 0.11	15.66 ± 0.58	9.71 ± 0.36	This study
		*Plasmodium spp*	*E.coli*	0.25 ± 0.03	-	-	Sangolgi et al. (2016b)
PGK	Disodium phosphoglycerate 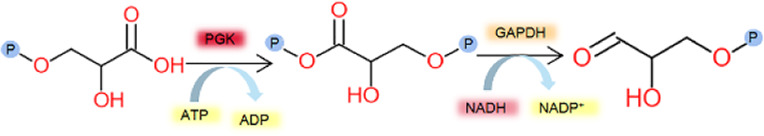	*C.glutamicum*	*yeast*	0.94 ± 0.15	67.84 ± 2.68	4.95 ± 1.96	This study
		*C*. *glutamicum*	*E.coli*	0.26	220.00	191.00	Reddy and Wendisch (2014)
PGMA	3-Phosphoglycerate 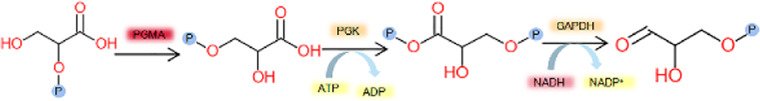	*C.glutamicum*	*E.coli*	4.44 ± 0.32	43.16 ± 2.21	20.45 ± 1.05	This study
		*S.japonicum*	*E.coli*	3.78	-	5.26	Guo (2010)
ENO	2- Phosphoglycerate 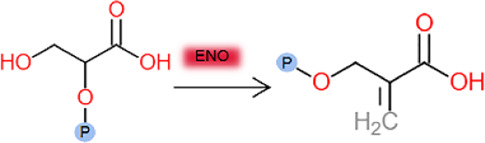	*C.glutamicum*	*yeast*	0.06 ± 0.01	21.31 ± 1.16	0.44 ± 0.02	This study
		*T*. *annulata*	*E.coli*	0.11	-	37.00	Cayir et al. (2014b)
ZWF	NADP^+^ 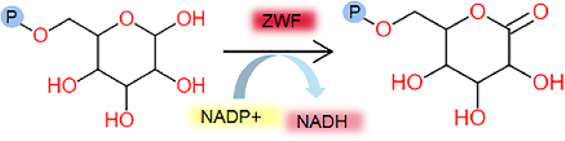	*C.glutamicum*	*E.coli*	0.46 ± 0.04	0.51 ± 0.04	0.50 ± 0.04	This study
		*H*. *pylori*	*E.coli*	0.012	-	70.00	Ortiz-Ramirez et al. (2022)
	glucose 6-phosphate 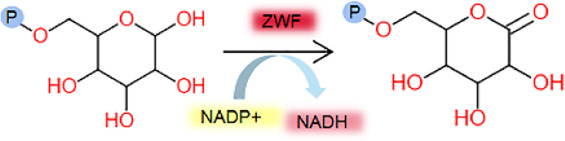	*C.glutamicum*	*E.coli*	1.61 ± 0.15	0.96 ± 0.04	0.94 ± 0.04	This study
		*H*.*pylori*	*E.coli*	0.075	-	70.00	Ortiz-Ramirez et al. (2022)
RPI	D- ribulose-5-phosphate 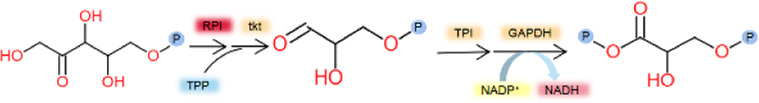	*C.glutamicum*	*E.coli*	0.42 ± 0.06	11.18 ± 0.74	3.39 ± 0.22	This study
RPE	D-ribulose-5-phosphate 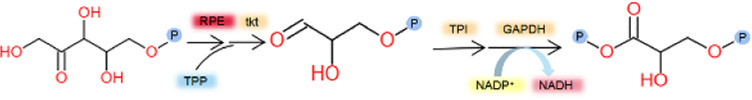	*C.glutamicum*	*E.coli*	2.81 ± 1.25	429.94 ± 53.08	178.70 ± 22.16	This study
TKT	E- xylulose-5-phosphat 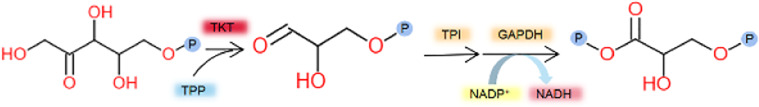	*C.glutamicum*	*E.coli*	0.47 ± 0.04	1.97 ± 0.12	2.50 ± 0.15	This study
		*M*. *tuberculosis*	*E.coli*	0.030	-	-	Shcherbakova et al. (2022)
	D- ribose-5-phosphate 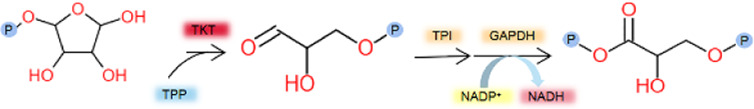	*C.glutamicum*	*E.coli*	1.13 ± 0.29	3.07 ± 0.48	3.91 ± 0.61	This study
		*M*. *tuberculosis*	*E.coli*	0.13	-	-	Shcherbakova et al. (2022)
TAL	d-sedoheptulose-7-phosphate 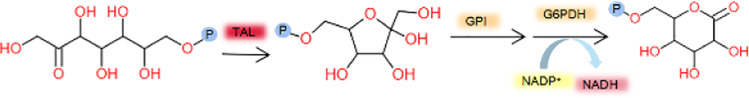	*C.glutamicum*	*E.coli*	0.82 ± 0.02	9.42 ± 0.18	6.20 ± 0.12	This study
		*E*.*coli K-12*	*E.coli*	0.28	-	-	Sprenger et al. (1995)
	glyceraldehyde-3-phosphate 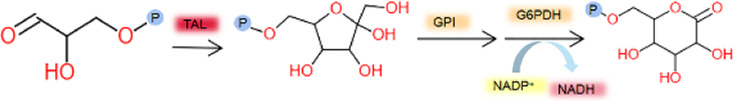	*C.glutamicum*	*E.coli*	0.29 ± 0.08	6.44 ± 0.71	4.24 ± 0.47	This study
		*E*.*coli K-12*	*E.coli*	0.038	-	-	Sprenger et al. (1995)

“-“, not recorded.

The enzymes GLK, GPI, TPI, GAPDH, PGK, PMGA and ENO correspond to the substrates glucose/ATP, D-fructose-6-disodium phosphate hydrate, dihydroxyacetone phosphate, dihydroxyacetone phosphate, glyceraldehyde triphosphate, disodium phosphoglycerate, 3-phosphoglycerate, and 2-phosphoglycerate with *K*_m_ values of 4.6 ± 0.18 mM/0.81 ± 0.08 mM, 0.57 ± 0.13 mM, 5.43 ± 0.16 mM, 1.47 ± 0.11 mM, 0.94 ± 0.15 mM, 4.44 ± 0.32 mM, and 0.06 ± 0.01 mM, respectively.

The enzymes ZWF, GND, RPI, RPE, TKT, and TAL correspond to the substrates NADP/glucose-6-phosphate, D-ribulose-5-phosphate, D-xylulose-5-phosphat/D-ribulose-5-phosphate and D-sedoheptulose-7-phosphate/glyceraldehyde-3-phosphate, respectively, with *K*_m_ values of 0.46 ± 0.04 mM/1.61 ± 0.15 mM, 0.42 ± 0.06 mM, 2.81 ± 1.25 mM, 0.47 ± 0.04 mM/1.13 ± 0.29 mM, 0.82 ± 0.02 mM/0.29 ± 0.08 mM, respectively (Table 1).

Compared with previous studies, more kinetic data of enzymes associated with EMP and HMP pathways were obtained under the same conditions. This is crucial because previous research reports indicate that powerful and potential metabolic network modeling requires accurate quantitative information about enzymatic reaction rates obtained under consistent experimenting methods or conditions (Tohsato et al., 2013). In this research, twelve enzymes kinetics parameters were achieved about EMP and HMP pathway from *C.glutamicum*, which provides a significant meaningful data basis for modeling the central metabolic pathway based on enzyme kinetics. At the same time, the measurement conditions for these parameters are essentially the same, which reduces the error caused by data obtained from different laboratories and measurement conditions in the process of the metabolic modeling in previous studies.

## 4 Discussion and conclusion

### 4.1 Analysis and prediction of rate limiting enzymes based on *K*_*m*_ value

*V*_max_ represents the maximum rate of the reaction under the given condition, while *K*_m_ is the substrate concentration at half the maximum reaction rate (Barenholz et al., 2017). The *K*_m_ value is often used to determine the affinity of an enzyme for its substrate and can indicate the rate-limiting step in a metabolic pathway. Enzymes with higher *K*_m_ values may have a lower affinity for their substrates, and therefore, in a series of enzyme chain reactions, the Km value of the enzyme will help to identify the rate limiting step in this reaction. In this study, the higher *K*_m_ values of GLK, GAPDH, TPI, ZWF, and RPE enzymes compared to other enzymes in the EMP and HMP metabolic pathway suggest that these enzymes may have a lower affinity for their substrates and be rate-limiting in their respective pathway.

**FIGURE 7:**
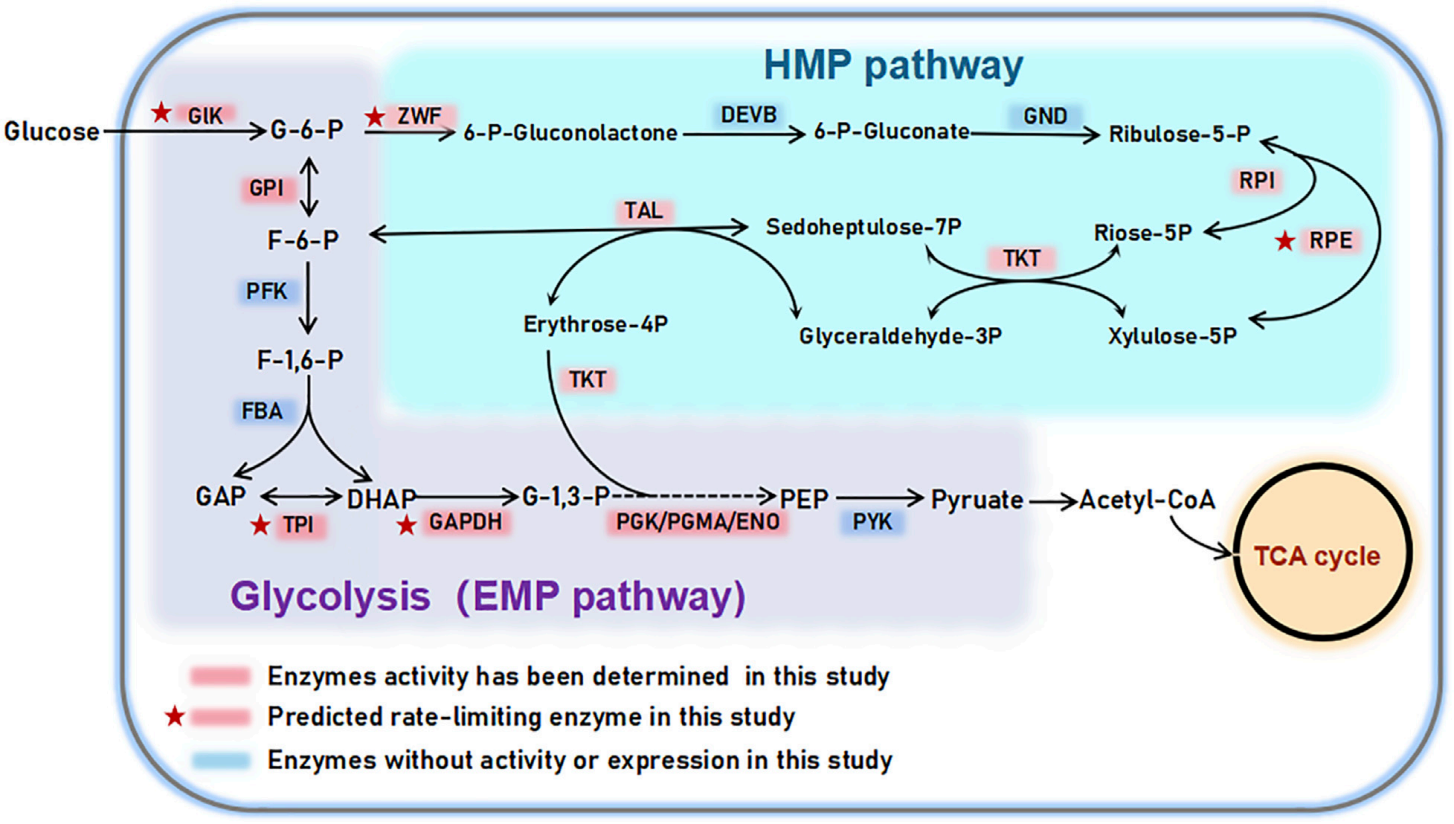
EMP and HMP pathway related enzymes determined in this study.

GLK, as the first enzyme in both the EMP and HMP pathway, catalyzes the conversion of glucose to glucose-6-phosphate. It is essential for glucose metabolism and considered as the rate-limiting enzyme in the procession of glucose metabolism (Milanes et al., 2019; Sternisha and Miller, 2019). TPI, located at the junction of the EMP and HMP pathways, is crucial for converting dihydroxyacetone phosphate (DHAP) and glycerol-3-phosphate (G3P) in the glycolytic pathway. It plays an essential role in the net production of ATP in glucose metabolism (Myers and Palladino, 2023; Schachner et al., 2022). ZWF is primarily responsible for converting glucose-6-phosphate (G6P) to 6-phosphate glucolactone, producing NADPH or NADH. This enzyme is critical for maintaining the redox balance within cells by generating NADPH, which is essential for various cellular processes (Gomez-Manzo et al., 2016). As a result, ZWF is recognized as a rate-limiting enzyme in the HMP metabolic pathway. Notably, RPI has a lower *K*_m_ value than RPE when using D-ribose 5-phosphate as a substrate, indicating that RPI is more effective than RPE in isomerizing ribose 5-phosphate. RPI and RPE involves the interconversion of ribose-5-phosphate and xylose-5-phosphate in the EMP and HMP pathways, which are important intermediates in nucleotide and cofactor biosynthesis. Therefore, there are also considered as key rate limiting enzyme on the HMP pathway. In summary, the enzymes identified by the *K*_m_ comparison in this study, which represent the rate-limiting step, are consistent with existing research reports, demonstrating the data availability of this study. It also provides valuable insights into the role and importance of GLK, TPI, ZWF, RPI and RPE enzymes in the EMP and HMP metabolic pathways (Figure 7).

### 4.2 Analysis of metabolic regulation based on enzyme activity

*K*_cat_ is the turnover number of enzyme, representing the number of substrate molecules converted to product per enzyme molecule per unit time. The *K*_cat_/*K*_m_ ratio, also known as the specificity constant, is a measure of enzyme efficiency. A higher *K*_cat_/*K*_m_ values indicates a higher catalytic efficiency and enzyme activity (Davidi et al., 2016; Niu et al., 2022). In this study, the *K*_cat_/*K*_m_ values of GPI and RPE were relatively high, indicating higher catalytic efficiency. On the other hand, GLK, ZWF, and TKT had relatively low *K*_cat_/*K*_m_ values, suggesting that they are less efficient in catalyzing reactions and have a lower catalytic activity. These enzymes should be regulated during the metabolic process to maintain cellular metabolic balance.

Genome-scale metabolic modeling studies show that upregulating ZWF, which is contributes to NADPH production, to increase isobutanol production by *C.glutamicum*. Conversely, downregulating enzymes like PGK was identified as a strategy for overproduction of 1,2-propylene glycol in (Zhang et al., 2017). On the other hand, overexpression of GAPDH can significantly improve glucose utilization and enhance shikimic acid production (Kogure et al., 2016). Mutation in enzymes, such as ZWF and GND, increase the supply of NADPH from the HMP pathway, resulting in improved methionine yield (Li et al., 2016). Indeed, in metabolic networks, overexpression of rate-limiting enzymes can be a valuable strategy to enhance the flux through specific metabolic pathways, which plays a crucial role in maintaining stable metabolic flux and minimizing the consumption of intermediate metabolites.

The use of enzyme kinetics parameters for intracellular metabolism flow analysis (MFC) and metabolic network modeling is very effective for analyzing microbial metabolic pathways and formulating optimal design and operation strategies for fermentation (Oshiro et al., 2009). Moreno-Sánchez R. established the first kinetic model of glycolytic metabolism through determined kinetic parameters for five enzymes and their reversal reactions, and predicted flux control by 3-phosphoglycerate mutase and hexokinase under conditions of low substrate and product concentrations (Moreno-Sanchez et al., 2008). By combining experimental enzyme characterizations with mathematical modeling approaches, Kuschmierz L. were able to design and optimize enzyme cascades and whole-cell biocatalysts for efficient d-xylose metabolism (Kuschmierz et al., 2022). Oshiro M. created a kinetic simulation model for acetone-butanol-ethanol fermentation and uncovered several important metabolic pathways that contribute to higher butanol yield (Oshiro et al., 2009). Apparently, cellular metabolic model construction, along with techniques like MFA, provides valuable insights into the balance of metabolic pathway fluxes and aids in the identification of rate-limiting steps. However, one of the challenges in developing kinetic model is the lack of comprehensive experimental data sets that can be used for parameterization, and gathering a complete set of kinetic parameters for all the enzymes and reactions in a metabolic network is a complex task.

In this study, 17 enzymes associated with the EMP and the HMP pathway were cloned from *C.glutamicum* ATCC 13032 and expressed in *E.coli* BL21 and *P.pastoris* X33 strains. By determining and calculating kinetic parameters such as *K*_m_, *K*_cat_, and *V*_max_ values of 12 enzymes, a systematic and complete characterization of their enzymes activities was achieved for the first time. These kinetic parameters of enzymes enable the prediction of key enzymes and rate-limiting steps within the metabolic pathway, as well as support the construction of a metabolic network model for important metabolic pathways in *C.glutamicum*. Such analyses and models aid in understanding the metabolic behavior of the organism and can guide the efficient production of high-value chemicals using *C.glutamicum* as a host.

## Data Availability

The datasets presented in this study can be found in online repositories. The names of the repository/repositories and accession number(s) can be found below: https://www.ncbi.nlm.nih.gov/, GCF_000011325.1.
