# Supplementary material for: Characterization of the enzyme kinetics of EMP and HMP pathway in Corynebacterium glutamicum: reference for modeling metabolic networks
Source: Front Bioeng Biotechnol. 2023 Nov 28;11:1296880. doi: 10.3389/fbioe.2023.1296880 (PMC10713844; doi:10.3389/fbioe.2023.1296880)
Supplement: Supplementary file 1 [file DataSheet1.PDF]

Supplementary Material

Characterization of the enzyme kinetics of EMP and HMP pathway in *Corynebacterium glutamicum*: reference for modeling metabolic networks

Liu Yang[1], Junyi Li[1], Yaping Zhang[1], Linlin Chen[1], Zhilin Ouyang[1], Daocheng Liao[1], Fengguang Zhao[1,2*] Shuangyan Han[1*]

1 Primers were amplified from the genomic DNA of *C.glutamicum* ATCC13032 about EMP and HMP pathway related enzyme genes.

Table S1. Primers used for *E.coli* BL21 expression plasmid construction.

Name	Sequence(5′-3′)	Gene
E1-glk-F	GCCATCATCATCATCATCACATGCCACAAAAACCGGC	Cgl2185
E1-glk-R	TTAGCAGCCGGATCTCACTAGTTGGCTTCCACTACAG	
E2-gpi-F	GCCATCATCATCATCATCACATGGCGGACATTTCGACCAC	Cgl0851
E2-gpi-R	TTAGCAGCCGGATCTCACTACCTATTTGCGCGGTACCA	
E3-pfk-F	GCCATCATCATCATCATCACATGCGAATTGCTACTCTCACG	Cgl1250
E3-pfk-R	TTAGCAGCCGGATCTCACTATCCAAACATTGCCTGGGC	
E4-fba-F	GCCATCATCATCATCATCACATGCCTATCGCAACTCCCG	Cgl2770
E4-fba-R	TTAGCAGCCGGATCTCATTACTTAGAGGTGGTCTTTCCAAC	
E5-tpi-F	GCCATCATCATCATCATCACATGGCACGTAAGCCACTTATC	Cgl1586
E5-tpi-R	TTAGCAGCCGGATCTCATTAAGCAACGCTCGCAGCG	
E6-gapdh-F	GCCATCATCATCATCATCACGATACCATTCGTGTTGGTAT	Cgl0937
E6-gapdh-R	TTGTTAGCAGCCGGATCTCATTAGAGCTTGGAAGCTACGA	
E7-pgk-F	GCCATCATCATCATCATCACATGGCTGTTAAGACCCTCAAGG	Cgl1587
E7-pgk-R	TTAGCAGCCGGATCTCATTACTGAGCGAGAATTGCAACG	
E8-pgma-F	GCCATCATCATCATCATCACATGACTAACGGAAAATTGATTCTTCTTCG	Cgl0402

	TC	
E8-pgma-R	TTAGCAGCCGGATCTCACTACTTATTACCCTGGTTTGCTACTGC	
E9-eno-F	GCCATCATCATCATCATCACGTGGCTGAAATCATGCACGT	Cgl0974
E9-eno-R	TTGTTAGCAGCCGGATCTCATTAGCCCTGAAAGCGTGGGA	
E10-pyk-F	GCCATCATCATCATCATCACATGGGCTGGGATAGACGAAC	Cgl2089
E10-pyk-R	TTGTTAGCAGCCGGATCTCATTAGAGCTTGCAATCCTTG	
H1-zwf-F	GCCATCATCATCATCATCACGTGAGCACAAACACGACCCC	Cgl1576
H1-zwf-R	TTGTTAGCAGCCGGATCTCATTATGGCCTGCGCCAGGTGT	
H2-devB-F	GCCATCATCATCATCATCACATGGTTGATGTAGTACGCGC	Cgl1578
H2-devB-R	TTGTTAGCAGCCGGATCTCATTAGAGATTTCCTGCAGCAT	
H3-gnd-F	GCCATCATCATCATCATCACATGCCGTCAAGTACGATCAA	Cgl1452
H3-gnd-R	TTGTTAGCAGCCGGATCTCATTAAGCTTCAACCTCGGAGC	
H4-rpi-F	GCCATCATCATCATCATCACATGCGCGTATACCTTGGAGC	Cgl2423
H4-rpi-R	TTGTTAGCAGCCGGATCTCATTATTCGTTAGGAACGACAG	
H5-rpe-F	GCCATCATCATCATCATCACATGGCACAACGTACTCCACT	Cgl1598
H5-rpe-R	TTGTTAGCAGCCGGATCTCATTACTGCGCGAGTGCTCGCA	
H6-tkt-F	GCCATCATCATCATCATCACTTGACCACCTTGACGCTGTC	Cgl1574
H6-tkt-R	TTGTTAGCAGCCGGATCTCATTAACCGTTAATGGAGTCCT	
H7-tal-F	GCCATCATCATCATCATCACATGTCTCACATTGATGATCT	Cgl1575
H7-tal-R	TTGTTAGCAGCCGGATCTCACTACTTCAGGCGAGCTTCCA	

Table S2. Primers used for *P.pastoris* X33 expression plasmid construction.

Name	Sequence(5′-3′)	Gene
E3-pfk-F′	AGAAAAGAGAGGCTGAAGCTGAATTCCGAATTGCTACTCTCACGTC	Cgl1250
E3-pfk-R′	GTTTTTGTTCTAGAAAGCTGGCGGCCGCTCCAAACATTGCCTGGGCAG	
E4-fba-F′	AGAAAAGAGAGGCTGAAGCTGAATTCCCTATCGCAACTCCCGAGGT	Cgl2770
E4-fba-R′	GAGTTTTTGTTCTAGAAAGCTGGCGGCCGCCTTAGAGGTGGTCTTTCC	
E7-pgk-F′	GCCATCATCATCATCATCACATGGCTGTTAAGACCCTCAAGG	Cgl1587

E7-pgk-R′	TTAGCAGCCGGATCTCATTACTGAGCGAGAATTGCAACG	
E9-eno-F′	AGAAAAGAGAGGCTGAAGCTGAATTCGTGGCTGAAATCATGCACGT	Cgl0974
E9-eno-R′	GAGTTTTTGTTCTAGAAAGCTGGCGGCCGCGCCCTGAAAGCGTGGGAATG	
H3-gnd-F′	AGAAAAGAGAGGCTGAAGCTGAATTCCCGTCAAGTACGATCAATAAC	Cgl1452
H3-gnd-R′	GAGTTTTTGTTCTAGAAAGCTGGCGGCCGCAGCTTCAACCTCGGAGCGGTC	

2 Enzyme activity assays

Unless otherwise stated, PBS (NaCl 137mM, KCl 2.7mM, Na_2HPO_4 10mM, KH_2PO_4 2mM, pH 7.4) was used as the buffer solution, the reaction temperature was 37℃, the reaction volume was 100μl, and fit to the Michaelis-Menten equation in Origin 9.0 software.

Glucokinase (GLK, EC: 2.7.1.2): The activity of GLK was determined in triplicate using a coupled reaction. The reaction mixture contains 100 mM PBS buffer, $MgCl_2$ 0.5 mM, $NAD(P)^+$ 0.4 mM, glucose-6-phosphate dehydrogenase 2 U/mL, ATP 0.02-2.5 mM, or glucose 0.2-50 mM, reaction 10 min at 37℃, the production of ADP at 340 nm was measures (Milanes et al.,2019).

Glucose-6-phosphate isomerase (GPI, EC:5.3.1.9): The activity of GPI was measured with the spectrophotomer in a coupled assay, with NAD(P)H formation measured at OD340 nm . The reaction mixture contained 100 mM PBS buffer, 1.25 mM $NAD(P)^+$, 0.2-12 mM fructose 6-phosphate, and 25 U glucose 6-phosphate dehydrogenase(Mathur et al.,2005;Rudolph et al.,2004).

6-phosphofructokinase (PFK, EC:2.7.1.11): The activity of PFK was measured spectrophotometrically by coupling with the auxiliary enzymes pyruvate kinase (PK) and lactate dehydrogenase (LDH). PFK catalyzes fructose-6-phosphate and ATP to generate fructose-diphosphate and ADP. Pyruvate kinase and lactate dehydrogenase also catalyze NAD(P)H oxidation to in turn generate $NAD(P)^+$, and PFK activity can be reflected by measuring the rate of NAD(P)H removal at 340 nm. The assay mixture consisted of 100 mM PBS buffer, 2.0 mM $MgSO_4$, 5.0 mM KCl, 0.728 mM PEP, 10 mM F-6-P, 1.0 mM ATP, 0.25 mM NAD(P)H, 0.35 U PK, 0.5 U LDH, and appropriate amounts of PFK (Le SB et al.,2017).

Fructose-bisphosphate aldolase(FBA, EC:4.1.2.13): Conditions of measurement aldolase activity: 100 mM PBS buffer, 1 mM FBP, 10 mM EDTA, 2 mg/ml α-glycerophosphate dehydrogenase, 2 mg/ml triose phosphate isomerase, 100 μg/ml BSA, 0.15 mM NAD(P)H and 10 μl purified enzyme extract; the change in NAD(P)H at 340 nm was determined using a spectrophotometer (Dawson et al.,2013).

Triosephosphate isomerase(TPI, EC:5.3.1.1): The activity of TPI was measured in the direction of conversion of Dihydroxyacetone phosphate (DHAP) conversion to glyceraldehyde 3-phosphate (GAP). The reaction was coupled with 100 mM dibasic sodium phosphate, 100 mM sodium dihydrogen phosphate, 0.2-20 mM dihydroxyacetone phosphate, 5mM $NAD(P)^+$, TPI 10 U/ml, monitored the change in NAD(P)H concentration at 340 nm(Mathur et al.,2006).

Glyceraldehyde 3-phosphate dehydrogenase(GAPDH, EC:1.2.1.12): GAPDH catalyzes the conversion of glyceraldehyde-3-phosphate to 1,3-bisphosphoglycerate in conjunction with the

reduction of $NAD(P)^+$. The reaction mixture contains 100 mM PBS buffer, $MgCl_2$ 0.5 mM, $NAD(P)^+$ 5 mM, GAPDH 7.59 μg/ml, glyceraldehyde 3-phosphate 0.05-5 mM, and monitors the change in NAD(P)H concentration at 340 nm (Sangolgi et al.,2016).

Phosphoglycerate kinase (PGK, EC:2.7.2.3): The activity of PGK was determined using a coupled reaction. The reaction mixture contains 100 mM PBS buffer, $MgCl_2$ 4 mM, $MgSO_4$ 5 mM, EDTA 1 mM, DTT 1 mM, NAD(P)H 0.2 mM, ATP 1 mM, GAPDH 4 U/mL, D-3-phosphoglyceric acid disodium salt 0.05-5 mM, and monitor the change in NAD(P)H concentration at 340 nm (Shang et al.,2021).

Phosphoglycerate mutase (PGMA, EC:5.4.2.11): The activity PGMA was determined using a coupled reaction. The reaction mixture consisted of 100 mM PBS buffer, $MgCl_2$ 4 mM, enolase 5 U/mL, 3-phospho-D-glycerate 0.5-10 mM, and the change of NAD(P)H concentration was measured at 340 nm (Guo,2010).

Enolase (ENO, EC:4.2.1.11): The of activity ENO was measured from the formation of PEP from 2-PGA by observing the increase in absorbance PEP at 240 nm. The reaction mixture contained 100 mM PBS buffer, 1.5 mM $MgCl_2$, 0.01-2 mM 2-PGA (Cayir et al.,2014).

Pyruvate kinase (PYK,EC:2.7.1.40): The activity of PYK was measured by an LDH coupled enzyme assay, The reaction mixture contained 100 mM PBS buffer, 100 mM KCl, 5 mM $MgCl_2$, 1 mM ADP, 0.5 mM PEP, 0.2 mM NADH, 8 U of LDH, and the change of NAD(P)H concentration was measured at 340 nm (Wiese et al.,2021).

Glucose-6-phosphate dehydrogenase (ZWF， EC:1.1.1.49/1.1.1.363): Glucose-6-phosphate and $NAD(P)^+$ are formed by the action of glucose-6-phosphate dehydrogenase to produce gluconic acid 6-phosphate and NAD(P)H, the latter having an absorption maximum at 340 nm. The reaction mixture contained 100 mM PBS buffer, 10 mM $MgCl_2$, 0.01-1 mM $NAD(P)^+$, 200 mM potassium glutamate or 0.1-4 mM glucose 6-phosphate. One unit of enzyme activity was defined as 1 μmol NAD(P)H elevated/min mg/protein (Hou et al.,2006).

6-phosphogluconolactonase (DEVB, EC:3.1.1.31): The activity of DEVB was measured at 340 nm: 100 mM PBS buffer; $NAD(P)^+$ 0.6 mM; 6-phosphogluconolactone 1.0 mM; 6-phosphate dehydrogenase 5 U/ml. After adding the appropriate amounts of 6-phosphogluconolactonase enzyme to the cuvette, measure the absorbance change at 340 nm.

Gluconate 6-phosphate dehydrogenase (GND, EC:1.1.1.44): The activity of GND was assayed by monitoring the change of $NAD(P)^+$ at 340 nm. the reaction mixture contained 100 mM PBS buffer, 100 μM $NAD(P)^+$ and varying concentrations of GND, 10 mM $MgCl_2$, and 0.5 mM EDTA (Haeussler et al.,2018).

Ribose-5-phosphate isomerase and ribulose-5-phosphate 3-epimerase (RPI and RPE, EC:5.3.1.6/EC:5.1.3.1): RPI and RPE enzyme activity was determined using a coupled reaction. The reaction mixture contained 100 mM PBS buffer, 0.24 mM $MgCl_2$, 0.01 mM thiamine pyrophosphate (TPP), 1 mM NAD(P)H, 3 U 3-phosphate-glycerol dehydrogenase, 10 U triose phosphate isomerase, 1 U transketolase, 0.05-1 mM D-ribulose-5-phosphate (Ru-5-P) and the appropriate amount of enzymes. One unit of enzyme activity was defined as 1 μmol NAD(P)H decreased/min mg/protein.

Ru-5-P →(RPI/RPE)→R-5-P

R-5-P →(TKT)→GAP+S-7-P

GAP⇌ (TPI)⇋ DHAP

DHAP + NAD(P)H→(GAPDH)→G-3-P+NAD(P)$^+$

Transketolase (TKT, EC:2.2.1.1): The activity of TKT was determined using a coupled reaction. The reaction mixture contained 100 mM PBS buffer, 0.24 mM $MgCl_2$, 0.01 mM TPP, 1 mM NAD(P)H, 3 U 3-phosphate-glycerol dehydrogenase, 10 U triose phosphate isomerase, 0.05-1 mM D-ribose-5-phosphate (R-5-P), or 0.05-1 mM D-xylulose-5-phosphate (Xu-5-P). One unit of enzyme activity was defined as 1 μmol NAD(P)H decreased/min mg/protein.

Xu-5-P /R-5-P →(TKT)→GAP+S-7-P

GAP⇌(TPI)⇋DHAP

DHAP+ NADH→(GAPDH)→G-3-P+NAD(P)$^+$

Transaldolase (TAL, EC:2.2.1.2): The activity of TAL was determined using coupled reaction. The reaction mixture contained 100 mM PBS buffer, 10 U 6-phosphate-glucose isomerase, 3U 6-phosphate-glucose dehydrogenase, 0.4 mM NAD(P)$^+$, 0.05-1 mM D-sedoheptulose-7-phosphate (S-7-P), 0.1-1 mM glyceraldehyde-3-phosphate (G-3-P). One unit of enzyme activity was defined as 1 μmol NAD(P)H elevated/min mg/protein (Tan et al.,2016).

S-7-P/G-3-P→(TAL) →F-6-P

F-6-P →(GPI)→G-6-P

G-6-P + NAD(P)$^+$→(ZWF)→6-PGA + NAD(P)H

References:

Cayir, E., Erdemir, A., Ozkan, E., Topuzogullari, M., Bolat, Z. B., and Akat, A., et al. (2014). Cloning of intron-removed enolase gene and expression, purification, kinetic characterization of the enzyme from theileria annulata. *Mol. Biotechnol.* 56(8)

Dawson, N. J., Biggar, K. K., Storey, K. B. (2013). Characterization of fructose-1,6-bisphosphate aldolase during anoxia in the tolerant turtle, trachemys scripta elegans: an assessment of enzyme activity, expression and structure. *PLoS One*. 8(7), e68830. doi: 10.1371/journal.pone.0068830

Guo, F. (2010) Studies on sjpgam of schistosoma japonicum. master. [Beijing]: Chinese Academy of Agricultural Sciences.

Haeussler, K., Fritz-Wolf, K., Reichmann, M., Rahlfs, S., Becker, K. (2018). Characterization of plasmodium falciparum 6-phosphogluconate dehydrogenase as an antimalarial drug target. *J. Mol. Biol.* 430(21), 4049-4067. doi: 10.1016/j.jmb.2018.07.030

Hou, F., Huang, J., Lu, J., Wang, Z., Zhang, H. (2006). Isolation and expression analysis of plastidic glucose-6-phosphate dehydrogenase gene from rice (oryza sativa l.). *Yi chuan xue bao = Acta genetica Sinica*. 33(5)

Le SB, Heggeset, T., Haugen, T., Naerdal, I., Brautaset, T. (2017). 6-phosphofructokinase and ribulose-5-phosphate 3-epimerase in methylotrophic bacillus methanolicus ribulose monophosphate cycle. *Appl. Microbiol. Biotechnol.* 101(10), 4185-4200. doi: 10.1007/s00253-017-8173-0

Mathur, D., Ahsan, Z., Tiwari, M., Garg, L. C. (2005). Biochemical characterization of recombinant phosphoglucose isomerase of mycobacterium tuberculosis. *Biochem. Biophys. Res. Commun.* 337(2), 626-632. doi: 10.1016/j.bbrc.2005.09.092

Mathur, D., Malik, G., Garg, L. C. (2006). Biochemical and functional characterization of triosephosphate isomerase from mycobacterium tuberculosis h37rv. *FEMS Microbiol. Lett.* 263(2), 229-235. doi: 10.1111/j.1574-6968.2006.00420.x

Milanes, J. E., Suryadi, J., Abendroth, J., Van Voorhis, W. C., Barrett, K. F., and Dranow, D. M., et al. (2019). Enzymatic and structural characterization of the naegleria fowleri glucokinase. *Antimicrob. Agents. Chemother.* 63(5). doi: 10.1128/AAC.02410-18

Rudolph, B., Hansen, T., Schonheit, P. (2004). Glucose-6-phosphate isomerase from the hyperthermophilic archaeon methanococcus jannaschii: characterization of the first archaeal member of the phosphoglucose isomerase superfamily. *Arch. Microbiol.* 181(1), 82-87. doi: 10.1007/s00203-003-0626-4

Sangolgi, P. B., Balaji, C., Dutta, S., Jindal, N., Jarori, G. K. (2016). Cloning, expression, purification and characterization of plasmodium spp. Glyceraldehyde-3-phosphate dehydrogenase. *Protein. Expr. Purif.* 117, 17-25. doi: 10.1016/j.pep.2015.08.028

Shang, Y., Qi, J., Wang, Y., Li, H., Liu, X., and Wang, S., et al. (2021). Cloning, expression and enzymatic activity of mycoplasma synoviae phosphoglycerate kinase. *Chinese Journal of Animal Infectious Diseases*. 29(03), 71-79. doi: 10.19958/j.cnki.cn31-2031/s.2021.03.011

Tan, Z., Chen, J., Zhang, X. (2016). Systematic engineering of pentose phosphate pathway improves escherichia coli succinate production. *Biotechnol. Biofuels*. 9(1)

Wiese, E. K., Hitosugi, S., Loa, S. T., Sreedhar, A., Andres-Beck, L. G., and Kurmi, K., et al. (2021). Enzymatic activation of pyruvate kinase increases cytosolic oxaloacetate to inhibit the warburg effect. *Nat. Metab.* 3(7), 954-968. doi: 10.1038/s42255-021-00424-5
